# Development of a Genetically Engineered Bivalent Vaccine against Porcine Epidemic Diarrhea Virus and Porcine Rotavirus

**DOI:** 10.3390/v14081746

**Published:** 2022-08-09

**Authors:** Wan Li, Mingkai Lei, Zhuofei Li, Huimin Li, Zheng Liu, Qigai He, Rui Luo

**Affiliations:** 1 State Key Laboratory of Agricultural Microbiology, College of Veterinary Medicine, Huazhong Agricultural University, Wuhan 430070, China; 2 Key Laboratory of Preventive Veterinary Medicine in Hubei Province, The Cooperative Innovation Center for Sustainable Pig Production, Wuhan 430070, China; 3 Key Laboratory of Pesticide Chemical Biology of Ministry of Education, Hubei International Scientific and Technological Cooperation Base of Pesticide and Green Synthesis, International Joint Research Center for Intelligent Biosensing Technology and Health, College of Chemistry, Central China Normal University, Wuhan 430079, China

**Keywords:** porcine epidemic diarrhea virus, porcine rotavirus, recombinant vaccine, immunogenicity

## Abstract

Porcine epidemic diarrhea virus (PEDV) is an enteric coronavirus that causes acute diarrhea, vomiting, dehydration, and a high mortality rate in neonatal piglets. In recent years, PEDV has been associated with co-infections with other swine enteric viruses, including porcine rotavirus (PoRV), resulting in increased mortality among newborn piglets. In this paper, we developed a bivalent vaccine against PEDV and PoRV by constructing a recombinant PEDV encoding PoRV VP7 (rPEDV-PoRV-VP7). The recombinant virus was constructed by replacing the entire open reading frame 3 (ORF3) in the genome of an attenuated PEDV strain YN150 with the PoRV VP7 gene using reverse genetic systems. Similar plaque morphology and replication kinetics were observed in Vero cells with the recombinant PEDV compared to the wild-type PEDV. It is noteworthy that the VP7 protein could be expressed stably in rPEDV-PoRV-VP7-infected cells. To evaluate the immunogenicity and safety of rPEDV-PoRV-VP7, 10-day-old piglets were vaccinated with the recombinant virus. After inoculation, no piglet displayed clinical symptoms such as vomiting, diarrhea, or anorexia. The PoRV VP7- and PEDV spike-specific IgG in serum and IgA in saliva were detected in piglets after rPEDV-PoRV-VP7 vaccination. Moreover, both PoRV and PEDV neutralizing antibodies were produced simultaneously in the inoculated piglets. Collectively, we engineered a recombinant PEDV expressing PoRV VP7 that could be used as an effective bivalent vaccine against PEDV and PoRV.

## 1. Introduction

Porcine epidemic diarrhea virus (PEDV), an enteric coronavirus, causes a highly contagious diarrheal disease characterized by vomiting, diarrhea, dehydration, and a high mortality rate in neonatal piglets. Classical PEDV (G1) was first discovered in Europe in the early 1970s [1]. In 2010, a highly pathogenic PEDV variant of (G2) emerged in China [2,3], which spread rapidly to many North American, European, and Asian countries [4,5,6,7]. Since then, variant PEDV has become the major pathogen that causes diarrhea and high mortality in piglets, resulting in significant economic losses to the global pig industry [8,9,10].

PEDV, belonging to the *alphacoronavirus* genus within the family *Coronaviridae*, carries a single-stranded, positive-sense RNA of approximately 28 kb in length [11]. The PEDV genome contains seven open reading frames (ORFs) flanked by 5′- and 3′- untranslated regions (UTRs), in the order 5′-ORF1a/1b-S-ORF3-E-M-N-3′. ORF1a/1b occupies the 5′-proximal two-thirds of the genome and encodes replicase polyproteins pp1a and pp1b that are further processed by proteases into 16 non-structural proteins (NSPs). PEDV encodes four structural proteins: the spike (S), envelope (E), membrane (M), and nucleocapsid (N) [12]. The S glycoprotein is essential for binding to host cell receptors and facilitating membrane fusion and entry. Remarkably, the surface location of S protein makes it a prime target for neutralizing antibodies [13]. ORF3 gene, located between S and E gene, encodes the only accessory protein in PEDV. A previous study showed the ORF3 protein is involved in the viral lifecycle by binding to the S protein, but the ORF3 gene is not necessary for viral replication in vitro [14]. In our previous study, we constructed a recombinant PEDV by replacing the ORF3 gene with Nano-luciferase for the high-throughput screening of antiviral drugs [15]. Therefore, the ORF3 is an optimal site in the PEDV genome for the insertion of foreign genes.

In addition to PEDV, other swine enteric viruses, such as porcine rotavirus (PoRV), porcine deltacoronavirus (PDCoV), and transmissible gastro-enteritis virus (TGEV), are also able to cause diarrhea in piglets. Currently, the co-infection of PEDV with PoRV is most common in China [16,17,18]. Zhu et al. reported that the co-infection of PEDV and PoRV was considered the most severe form of piglet diarrhea with a detection rate of 16.16–25.23% in 2015–2016 in Northeast China. Consistently, the epidemiological surveys in Guangxi from 2017 to 2019 and in South-Central China from 2012 to 2022 showed that the co-infection of PEDV with PoRV became the dominant cause of diarrhea piglets [19,20,21,22]. Rotavirus (RV), a double-stranded RNA virus, often results in diarrhea-related illnesses in young children and animals worldwide [23,24,25,26]. The RV genome consists of 11 genome segments encoding six structural proteins and six NSPs. The outer capsid protein VP7 is a major immunogenic glycoprotein containing multiple neutralizing epitopes [27]. Group A rotavirus (RVA) is the most important rotavirus species causing acute dehydrating diarrhea in humans and animals [28,29]. In 1990, porcine RVA (PoRVA) first emerged in the United States. Subsequently, RVAs including G3, G4, G5, G9, and G11 genotypes were identified in pigs worldwide. In China, the G9 genotype is now the most prevalent genotype of PoRVA in swine [30,31,32]. In this paper, we construct a recombinant PEDV expressing the VP7 gene of a genotype G9 PoRVA strain and characterize its growth kinetics and VP7 expression in vitro. Furthermore, we evaluate the immunogenicity of this recombinant virus in piglets.

## 2. Materials and Methods

### 2.1. Cells, Viruses, and Antibodies

Vero and HEK-293T cells were cultured in Dulbecco’s modified Eagle’s medium (DMEM, Gibco, Thermo Fisher Scientific, Waltham, MA, USA) with 10% fetal bovine serum (FBS). MA104 cells were cultured in Minimum Essential Medium (MEM, Gibco, Thermo Fisher Scientific) containing 10% FBS. The cell-culture-adapted PEDV YN150 strain (GenBank accession number MZ581326) was obtained by serial passages of the high virulence PEDV YN1 (GenBank accession number KT021227) [33,34]. PEDV was cultured on Vero cells and grown in a maintenance medium containing DMEM with 15 μg/mL trypsin (Sigma) and 10% tryptose phosphate broth (TPB). The PoRV TM-a (GenBank accession number JF970186) was cultured on MA104 cells and grown in MEM with 5 μg/mL trypsin and 10% TPB. The anti-PEDV S mAb 4B2 were stored in our laboratory.

### 2.2. Preparation of the Monoclonal Antibody against VP7

The VP7 gene lacking the transmembrane region was amplified by RT-PCR and the PCR product was inserted into a prokaryotic expression vector pET-28a. The recombinant plasmid pET-28a-VP7 was expressed in *Escherichia coli* Rosetta (DE3) under IPTG induction at 37 °C for 5 h. After NI-NTA purification, five female BALB/C mice were immunized with the recombinant VP7 protein (His-VP7). Antibody-secreting spleen cells were isolated and fused with the myeloma using polyethylene glycol (PEG). After selection, one clone of hybridoma cells that secreted the monoclonal antibody (mAb) against VP7 was obtained and named 3H5.

### 2.3. Indirect Immunofluorescence Assay (IFA)

Cells were fixed with 4% paraformaldehyde for 15 min, permeabilized with 0.1% Triton X-100 (Sigma, St. Louis, MO, USA) for 10 min, and blocked with 5% bovine serum albumin (BSA) in PBS for 1 h. Subsequently, the cells were incubated with primary antibodies for 2 h, followed by Alexa Fluor 488 donkey anti-mouse IgG (Invitrogen) for 45 min. Finally, cells were stained with 4′,6-diamidino-2-phenylindole (Invitrogen) for 15 min, and the fluorescence signal was examined using a fluorescence microscope.

### 2.4. The Construction of a Recombinant PEDV Expressing PoRV VP7 Gene

The recombinant PEDV expressing PoRV VP7 (rPEDV-PoRV-VP7) was rescued based on the infectious cDNA clone of PEDV strain YN150 (icPEDV-YN150) as described previously [15]. Briefly, the ORF3 within icPEDV-YN150 was replaced by PoRV VP7 using the NEB Q5 site-directed mutagenesis kit (NEB, Ipswich, MA, USA) and ABclonal MultiF Seamless Assembly Mix (ABclonal, Wuhan, China), and confirmed by Sanger sequencing. After digestion, purification, and ligation, the full-length cDNA of rPEDV-PoRV-VP7 was assembled. rPEDV-PoRV-VP7 RNA was generated by in vitro transcription using mMESSAGE mMACHINE T7 Transcription Kit (Ambion). The full-length rPEDV-PoRV-VP7 RNA transcripts and PEDV N RNA transcripts were together electroporated into Vero cells at 450 V and 50 µF. At 12 h after electroporation, the cells were washed with PBS and cultured in DMEM with 15 μg/mL trypsin. Rescued rPEDV-PoRV-VP7 was harvested and further purified by plaque assay.

### 2.5. Virus Genome Sequencing

Viral RNAs were extracted from the Vero cells infected by rPEDV-PoRV-VP7 using TRIzol Reagent (Invitrogen, Waltham, MA, USA) and converted to cDNA by Transcriptor First Strand cDNA Synthesis Kit (Roche, Basel, Switzerland). The full-length genome of rescued PEDV was amplified and sequenced with primers described in our previous study [15].

### 2.6. Immunization

All the experimental and animal management procedures were approved by the Institutional Committee of the Huazhong Agriculture University for the Animal Experiments (HZAUSW-2022-0005), Wuhan, China. Ten 10-day-old piglets were purchased from a pig farm and were randomly divided into two groups (Mock-inoculated group and rPEDV-PoRV-VP7-inoculated group), which were housed in separated containments. Prior to immunization, rectal swabs from all piglets were confirmed negative for PEDV, PoRV, PDCoV, and TGEV by RT-PCR. Neutralizing antibodies against PEDV and PoRV were not detected in the sera of all piglets. After acclimation for 24 h, rPEDV-PoRV-VP7 was inoculated to the vaccinated group at a titer of 5 × 10^6^ TCID_50_/mL (2 mL oral administration and 2 mL intramuscular administration per piglet), while mock-infected piglets were inoculated with the same volume of DMEM medium as a negative control.

### 2.7. Sample Collection

Piglets were monitored daily for the presence of vomiting, diarrhea lethargy, and anorexia, and their weights were recorded weekly. The sera and oral swabs were collected from the vaccinated and unvaccinated piglets on days 0, 7, 14, 21, and 28. The sera were collected into a 1.5 mL centrifuge tube and stored at −80 °C until use. The oral swabs were immersed in 1 mL PBS and vortexed for 3 min. Then, the supernatants were harvested after centrifugation at 12,000 r/min for 10 min at 4 °C, and stored at −80 °C until use.

### 2.8. Expression of PEDV-S1

PEDV S1 gene was cloned into the pCAGGS expression vector with a mouse-Fc tag at the C-terminus (pCAGGS-mFc) and expressed in HEK-293T cells. The recombinant PEDV S1 protein (S1-mFc) was purified by Protein A affinity chromatography (GE healthcare, Protein A Sepharose™ CL-4B) and stored at −80 °C until use.

### 2.9. Enzyme-Linked Immunosorbent Assay (ELISA) Analysis

Titers of PEDV and PoRV antibodies in the serum and saliva were determined using an indirect ELISA with purified PoRV VP7 or PEDV S1 protein as the antigens. Briefly, ELISA plates were coated with purified His-VP7 or S1-mFc and blocked with 5% skimmed milk in PBS with 0.1% Tween 20 (PBST). For IgG detection, the serum samples were added after two-fold serial dilution (starting with a dilution of 1:100). For IgA detection, 100 μL oral swab samples were added to the plates. Then, the plates were incubated at 37 °C for 2 h. An HRP-conjugated goat anti-pig IgG or IgA (Bio-Rad, USA) antibody was added and incubated at 37 °C for 1 h, followed by color development using 3, 3′, 5, 5′-tetramethyl benzidine (TMB) substrate at 37 °C for 15 min. The reaction was stopped and the absorbance was measured at 450 nm.

### 2.10. Neutralizing Antibody Titration

The serum samples were inactivated at 56 °C for 30 min. After two-fold dilution, 50 μL of the serum dilutions were mixed with an equal volume of virus supernatant containing 200 TCID_50_ PoRV or PEDV, and incubated at 37 °C for 1 h. Vero or MA104 cells in 96-well plates were washed twice with PBS, covered with the serum–virus mixture in quadruplicates, and cultured in a maintenance medium supplemented with trypsin. Following incubation at 37 °C for 1 h, the mixtures were replaced by a 100 μL fresh maintenance medium. Cytopathic effects (CPE) were observed by microscope. The neutralizing antibody titer was determined as the highest serum dilution resulting in an infection reduction of 50%.

### 2.11. Statistical Analysis

The statistical analyses were performed using GraphPad Prism 6.0. The values of TCID_50_, weekly weight gain, IgG, IgA, and neutralizing antibody were analyzed by multiple *t*-tests. Viral growth kinetics were analyzed by one-way analysis of variance (ANOVA) followed by Tukey’s multiple-comparison test. * *p* < 0.05 was considered statistically significant and ** *p* < 0.01 was considered highly significant.

## 3. Results

### 3.1. The Preparation of Monoclonal Antibody (mAb) against PoRV VP7

**Figure 1:**
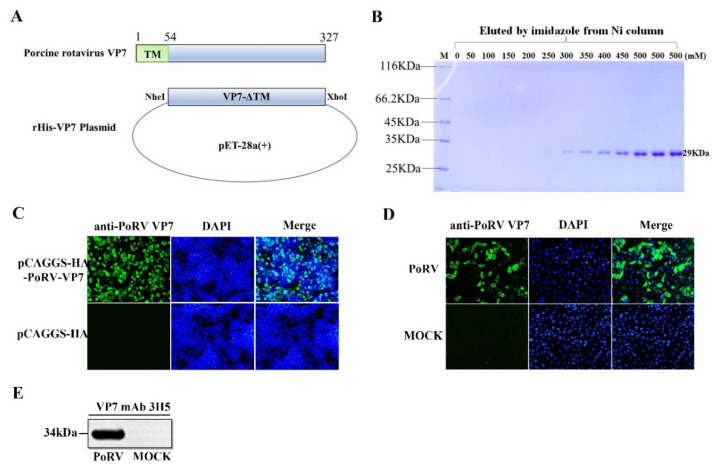
Preparation of monoclonal antibody against PoRV VP7. (**A**) Schematic diagram of the recombinant plasmid expressing PoRV VP7. (**B**) The SDS-PAGE analysis of the rHis-VP7 protein that was eluted by different concentrations of imidazole elution. (**C**) HEK-293T cells were transfected with the PoRV-VP7 expression plasmid. At 24 h post-transfection, VP7 expression was detected by IFA using mAb 3H5 (magnification, ×100). (**D**) MA104 cells were infected with 0.1 MOI PoRV. After 18 h post-infection, VP7 expression in PoRV-infected cells was examined by IFA using mAb 3H5 (magnification, ×100). (**E**) PoRV-infected MA104 cells was analyzed at 18 h post-inoculation by Western blotting using the anti-VP7 mAb 3H5.

In order to prepare the mAb against PoRV VP7, the VP7 gene lacking the TM domain was cloned into the pET-28a vector and expressed in *E. coil* (Figure 1A). After cell lysis and NI-NTA purification, the recombinant VP7 protein with His-tags eluted from Ni-affinity chromatography was analyzed by resolving on 12% SDS- PAGE. As shown in Figure 1B, the high purity VP7 protein was obtained with the expected molecular weight (MW) of approximately 29 kDa (Figure 1B). After immunization with the purified VP7 protein, splenocytes in mice were purified and fused with the myeloma to generate hybridomas. One clone of hybridoma cells secreting the mAb against VP7, named 3H5, was identified by the IFA analysis of VP7 protein expression in HEK-293T cells (Figure 1C). Then, the mAb 3H5 was used to detect VP7 expression in PoRV-infected MA104 cells by IFA and Western blotting. As shown in Figure 1D,E, specific signals were observed in the PoRV-infected cells, indicating that the mAb 3H5 could be used to detect PoRV VP7 expression.

### 3.2. Rescue of rPEDV-PoRV-VP7

**Figure 2:**
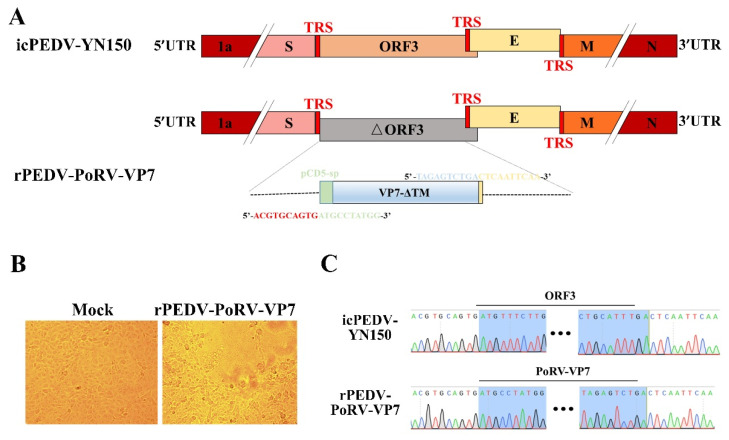
Rescue of rPEDV-PoRV-VP7. (**A**) Schematic representation of the cDNA clone of rPEDV-PoRV-VP7. (**B**) The CPE of rescued rPEDV-PoRV-VP7 in Vero cells was observed by microscope (magnification, ×100). (**C**) Identification of the ORF3 replacement with PoRV VP7 by whole-genome sequencing.

The recombinant PEDV expressing PoRV VP7 was engineered based on the infectious clone of an attenuated PEDV strain, YN150 [15]. As shown in Figure 2A, the ORF3 gene within the infectious clone of PEDV YN150 was replaced by the PoRV VP7 gene with the CD5 signal peptide (CD5-sp). After in vitro transcription, the full-length rPEDV-PoRV-VP7 RNA transcripts were electroporated into Vero cells, and PEDV-specific cytopathic effects (CPE) were observed at 20 h post-electroporation (Figure 2B). Then, the rescued virus was purified by plaque purification, and the complete genome of rPEDV-PoRV-VP7 was verified by sequencing. The sequencing results show that the ORF3 was successfully replaced with VP7 genes and no unwanted nucleotide change was found in the viral genome compared to the infectious cDNA clones (Figure 2C). It is noteworthy that the exogenous VP7 gene was successfully retained within the genome of rPEDV-PoRV-VP7 even after five passages (data not shown). Altogether, a recombinant PEDV encoding PoRV VP7 was successfully rescued.

### 3.3. Characterization of rPEDV-PoRV-VP7

**Figure 3:**
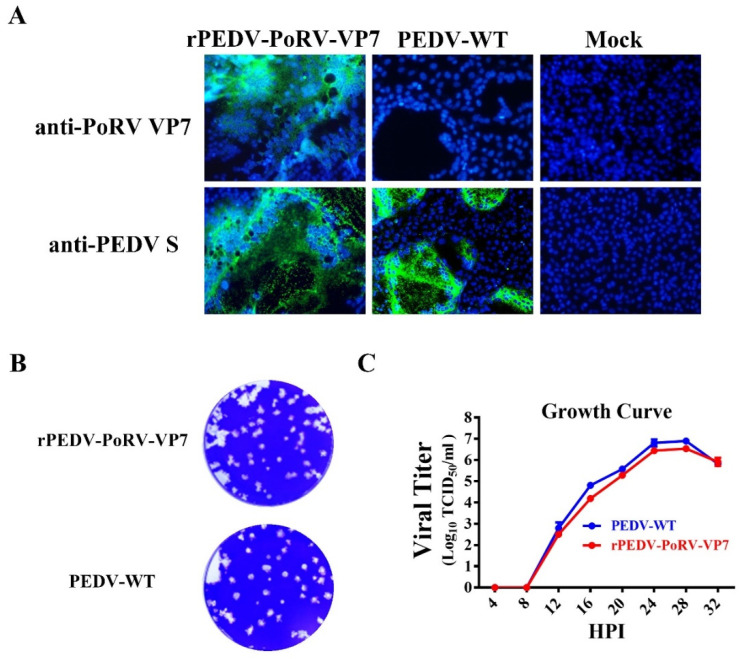
Characteristics of rPEDV-PoRV-VP7. (**A**) PoRV VP7 and PEDV S were detected in rPEDV-PoRV-VP7-, wild-type PEDV-, or mock-infected Vero cells by IFA (magnification, ×100), respectively. (**B**) Representative plaques of rPEDV-PoRV-VP7- or wild-type PEDV-infected Vero cells at 20 hpi. (**C**) Vero cells were infected with rPEDV-PoRV-VP7 or wild-type PEDV at an MOI of 0.01, and cell cultures were collected at indicated times to determine the viral titers by TCID_50_.

To further confirm the expression of VP7 in the recombinant virus genome, Vero cells were infected by rPEDV-PoRV-VP7 and examined by IFA with the mAb 3H5. As shown in Figure 3A, VP7-specific immunofluorescence was readily examined in rPEDV-PoRV-VP7-infected cells, indicating that the VP7 protein was successfully expressed. Meanwhile, the PEDV S-specific immunofluorescence signals were also detected, demonstrating the replication of rPEDV-PoRV-VP7 in Vero cells. To investigate whether the insertion of PoRV VP7 affects viral replication, we characterized the plaque morphology and replication kinetics of rPEDV-PoRV-VP7 in Vero cells. As shown in Figure 3B, rPEDV-PoRV-VP7 produced similar plaque morphology compared to the wild-type PEDV. The replication kinetics of rPEDV-PoRV-VP7 resembled that of wild-type PEDV, reaching a peak titer of 10^7^ TCID_50_/mL at 28 h post-infection (Figure 3C). These results suggest that the replacement of ORF3 with PoRV VP7 in PEDV YN150 did not affect viral proliferation and the PoRV VP7 could be effectively expressed in rPEDV-PoRV-VP7 genome.

### 3.4. Clinical Manifestations of the rYN150-PoRV-VP7-Inoculated Piglets

**Figure 4:**
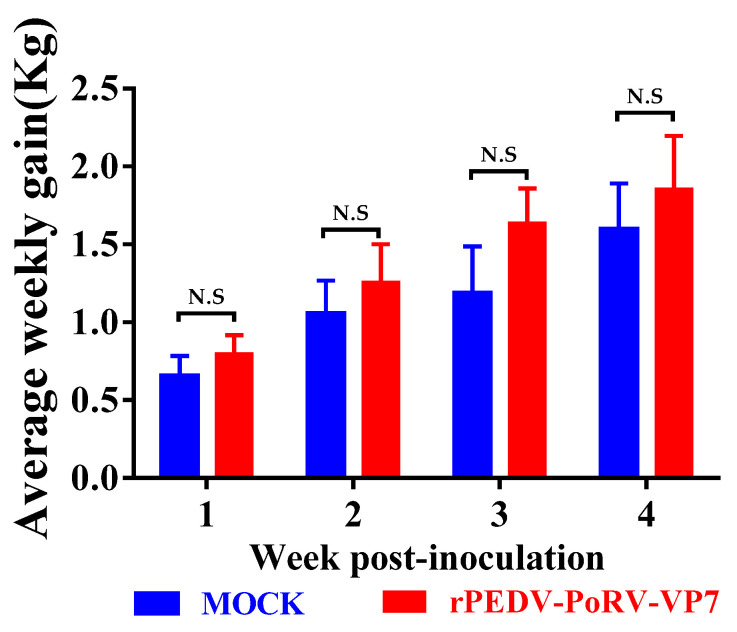
The weekly weight gain of rPEDV-PoRV-VP7-vaccinated piglets. Average weekly weight gain values are shown as the means ± SD. N.S, no significance.

Five 10-day-old piglets were inoculated with rPEDV-PoRV-VP7 to determine its immunogenicity and safety. Similar to the mock-inoculated group, rPEDV-PoRV-VP7-inoculated piglets did not display any clinical symptoms, such as vomiting, diarrhea, and dehydration. As expected, there was no significant difference in the average weekly weight gains between the rPEDV-PoRV-VP7- and mock-inoculated groups (Figure 4). These results demonstrate that rPEDV-PoRV-VP7 inoculation had no adverse effects on the health and growth of piglets.

### 3.5. IgA Levels in rPEDV-PoRV-VP7-Vaccinated Piglets

**Figure 5:**
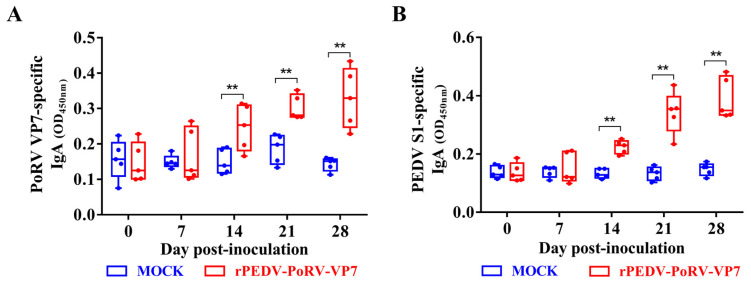
IgA levels in the rYN150-PoRV-VP7-vaccinated piglets. IgA antibodies against PoRV VP7 (**A**) and PEDV S1 (**B**) in oral swabs were collected from the rYN150-PoRV-VP7-inoculated piglets at 0, 7, 14, 21, and 28 DPI were measured by indirect ELISA using His-VP7 or S1-mFc. Mean ± SDs from five piglets inoculated or mock-inoculated with rYN150-PoRV-VP7. ** *p* < 0.01, as compared with the mock-inoculated group.

IgA is often used to measure mucosal immune against enterovirus infections [35]. To determine the IgA levels induced by rYN150-PoRV-VP7, the oral swabs from all piglets were collected at different times post-inoculation and detected by indirect ELISA using His-VP7 and S1-mFc as antigens, respectively. As shown in Figure 5A,B, the levels of anti-PoRV VP7 and anti-PEDV S IgA antibodies in saliva from rPEDV-PoRV-VP7-inoculated piglets were significantly increased from 14 to 28 days post-inoculation (DPI) compared with mock-inoculated piglets. These results suggest that rPEDV-PoRV-VP7 could effectively induce the specific IgA antibodies against PoRV and PEDV in piglets.

### 3.6. IgG Levels and Neutralizing Antibodies Induced by rPEDV-PoRV-VP7

**Figure 6:**
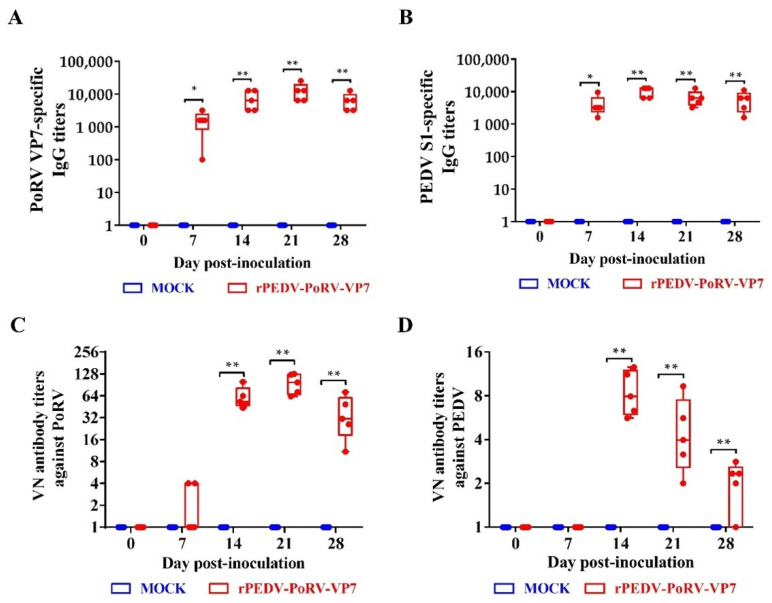
The titers of IgG and neutralizing antibody in serum from the rPEDV-PoRV-VP7-inoculated piglet. Levels of the anti-PoRV VP7-specific (**A**) and anti-PEDV S1-specific (**B**) IgG antibody. Neutralizing activity against PoRV (**C**) and PEDV (**D**) in serum was examined by viral neutralization assay in Vero cells. Each point represents the titer of an individual piglet. Data are shown as mean ± SD. * *p* < 0.05 and ** *p* < 0.01, as compared with the mock-inoculated group.

To measure the IgG levels induced by rPEDV-PoRV-VP7, the sera were collected from inoculated piglets at 0, 7, 14, 21, and 28 DPI, and measured by indirect ELISA. As shown in Figure 6A,B, the IgG level against PoRV VP7 and PEDV S1 in the serum from rPEDV-PoRV-VP7-inoculated piglets was significantly increased from 7 DPI and remained high up to the fourth week. Additionally, the PoRV VP7 antibody reached a peak at 21 DPI (Figure 6A), while PEDV S1 specific IgG antibody peaked at 14 DPI (Figure 6B). Next, we measured the neutralizing antibodies against PoRV and PEDV in the serum from the rYN150-PoRV-VP7-inoculated vaccinated piglets. We observed that neutralization antibody titers against PoRV were initially detected at 7 DPI and elevated to about 1:97 at 21 DPI (Figure 6C), while neutralization antibody titers against PEDV reached 1:8 at 14 DPI and dropped to approximate 1:2 at 28 DPI (Figure 6D).

## 4. Discussion

In recent years, the occurrences of PEDV-PoRVA co-infection increased rapidly in piglets, resulting in high mortality and massive economic losses to the pig industry [21,36,37]. The development of an effective bivalent vaccine against PEDV and PoRV is urgently required to prevent and control porcine viral diarrhea. Recently, Yin et al. constructed a recombinant DNA plasmid co-expressing the VP7 protein of PoRV and S protein and evaluated its humoral and cellular immune responses in mice. This plasmid DNA immunization was reported to induce considerable IFN-γ and IL-4 production in mice, as well as PoRV- and PEDV-specific IgG antibodies. However, the efficacy of the DNA vaccine in piglets against PEDV and PoRV was not undetermined [38]. Another report employs a *lactobacillus* delivery system to express the antigens of PEDV, PoRV, and TGEV. After the oral administration of the recombinant *lactobacillus*, the specific sIgA in nose swab and IgG in the serum against PEDV, PoRV, and TGEV were detected in immunized piglets. Additionally, this vaccine alleviated the clinical symptoms and reduced pathological intestinal injury in PEDV-infected piglets, but could not completely inhibit fecal shedding of PEDV [39]. A live recombinant viral vector vaccine is an attractive approach to prevent the co-infection of porcine enteroviruses. A previous study showed that TGEV was used as a coronavirus vector expressing the rotavirus VP7 protein that could provide partial protection against rotavirus-induced diarrhea in a murine model [40]. In this study, we used an attenuated PEDV strain, YN150, as a live virus vector for expressing the PoRV VP7 to develop a bivalent vaccine against PEDV and PoRV.

VP7, an outer capsid glycoprotein of rotavirus, is an ideal target for RV vaccine development. It possesses B- and T-cell antigenic determinants and induces protective immune responses in vivo [41]. Based on this information, the PoRV VP7 gene was inserted into the genome of PEDV YN150 to engineer a recombinant PEDV expressing the PoRV VP7 gene in this study. For PEDV, the ORF3 gene encodes the only accessory protein that is not essential for viral replication [42]. In our previous study, we have successfully expressed the exogenous NLuc gene by replacing the ORF3 gene of PEDV YN150 using a reverse genetic approach [15]. Therefore, the ORF3 of PEDV YN150 is the optimal site for the insertion of PoRV VP7. As expected, we found that the VP7 gene was stably expressed in the genome of PEDV YN150, and VP7 insertion had no impact on viral replication (Figure 3).

Signal peptide (SP), typically located at the N-terminus of nascent secreted protein, plays an essential role in protein secretion and glycosylation modifications [43]. A previous study demonstrated that SP has been used to enhance the immunogenicity of antigens by promoting protein expression and secretion [44]. For example, the addition of the murine IgGκ SP enhances the immunogenicity of vivax multistage vaccine by improving the CD4^+^ T-cell response and antibody avidity [45]. In this study, CD5 SP was added to the upstream of VP7 to increase VP7 immunogenicity. As expected, rPEDV-PoRV-VP7 produced a high level of PoRV VP7-specific mucosal IgA, plasma IgG, and neutralizing antibodies in piglets after inoculation (Figure 5 and Figure 6). Remarkably, sIgA is the predominant antibody at the mucosal surfaces of newborn piglets that play an indispensable role in combating enteric virus infection [46]. In this study, both PoRV VP7- and PEDV S-specific IgA antibodies were detected in the saliva from rYN150-PoRV-VP7-inoculated piglets, indicating that rPEDV-PoRV-VP7 could effectively induce mucosal immunity (Figure 5). In the future study, we will determine the protective effects of the bivalent vaccine against PEDV and PoRV using the highly virulent strains.

In summary, our study engineered a recombinant PEDV expressing the PoRV VP7 protein using the reverse genetic platform of an attenuated PEDV train, YN150. The recombinant virus, rPEDV-PoRV-VP7, showed similar replication kinetics to the parental virus in vitro. Of note, rPEDV-PoRV-VP7-inoculated piglets exhibited excellent immunogenicity, producing specific IgA, IgG, and neutralizing antibodies against PoRV and PEDV. Our findings provide a novel bivalent vaccine candidate for the prophylaxis of PEDV-PoRV co-infection.

## Data Availability

Data are contained within the article.
